# The direct cost of "Thriasio" school screening program

**DOI:** 10.1186/1748-7161-2-7

**Published:** 2007-05-14

**Authors:** Theodoros B Grivas, Elias S Vasiliadis, Christina Maziotou, Olga D Savvidou

**Affiliations:** 1 Orthopaedic Department, "Thriasio" General Hospital, G. Gennimata Av. 19600, Magoula, Attica, Greece

## Abstract

**Background:**

There is great diversity in the policies for scoliosis screening worldwide. The initial enthusiasm was succeeded by skepticism and the worth of screening programs has been challenged. The criticisms of school screening programs cite mainly the negative psychological impact on children and their families and the increased financial cost of visits and follow-up radiographs. The purpose of this report is to evaluate the direct cost of performing the school screening in a district hospital.

**Methods:**

A cost analysis was performed for the estimation of the direct cost of the "Thriasio" school-screening program between January 2000 and May 2006. The analysis involved all the 6470 pupils aged 6–18 years old who were screened at schools for spinal deformities during this period. The factors which were taken into consideration in order to calculate the direct cost of the screening program were a) the number of the examiners b) the working hours, c) the examiners' salary, d) the cost of transportation and finally e) the cost of examination per child.

**Results:**

During the examined period 20 examiners were involved in the program and worked for 1949 working hours. The hourly salary for the trainee doctors was 6.80 euro, for the Health Visitors 6.70 euro and for the Physiotherapists 5.50 euro in current prices. The cost of transportation was 32 euro per year. The direct cost for the examination of each child for the above studied period was calculated to be 2.04 euro.

**Conclusion:**

The cost of our school-screening program is low. The present study provides a strong evidence for the continuation of the program when looking from a financial point of view.

## 

"*It is better to prevent than to treat*" 

Ancient Greek saying

## Background

There is great diversity in the policies for scoliosis screening worldwide. The initial enthusiasm was succeeded by skepticism and the worth of screening programs has been challenged [1-4].

Scoliosis Research Society and the American Academy of Orthopaedic Surgeons continue to support the principle of school screening for scoliosis [5] and recommend screening girls at ages 11 and 13 years and screening boys once at 13 or 14 years [6]. US Preventive Service Task Force recommends against the routine screening of asymptomatic adolescents for idiopathic scoliosis [7]. The British Orthopaedic Association and the British Scoliosis Society conclude that it should not be a national policy to screen children for scoliosis routinely throughout the United Kingdom [8].

Criticism of school screening programs cite mainly the negative impact on children and their parents, because they produce anxiety, inconvenience, radiation exposure from follow-up roentgenograms, school missing for students and loss of working hours for parents for unnecessary follow up appointments [9]. Furthermore the financial cost of the screening programs, the cost of visits and the cost of follow-up radiographs has been implicated [10].

Two different school screening costs can be identified [11]; 1) *the direct cost *which is the cost of the screening program per se, or the cost which can directly be assigned to the program relatively easily with a high degree of accuracy; examiners' salaries, wages, materials, supplies, equipment, travel, consulting, printing, telephone, and photocopying and 2) *the indirect cost *which is the cost of false positive results, the follow up visits, the radiographs and the cost of brace treatment and/or surgery. In the indirect cost the hospital facilities and administrative costs could be included. In addition as an indirect cost can be recognized the financial and psychological impact of the screening procedure and the identification of scoliosis on the child and on his or her family, (see also the terminology of direct and indirect cost at the definitions section, at the end of the text).

The early detection of idiopathic scoliosis has been a major and growing commitment of orthopaedists since the early 1960s. Early detection implies early treatment and by that less surgery [11-13]. Thus, increased costs at an early stage may decrease later costs. The purpose of this report is to calculate the direct cost of performing the school screening in a district hospital.

## Methods

The school screening program took place at district schools (primary, secondary, high schools) around "Thriasio" Hospital in Western Attica, Greece.

School screening had to be set up on a district basis after obtaining permission from the local authorities, because such a program is not legislated in Greece. All the interested parties (parents, physicians, school staff, and nurses) were informed and when it was necessary, they were further educated by distribution of informative material and lectures. The cooperation of the screening staff with the parents, the pupils and the teachers is essential for the acceptance of this voluntary program and thus for a success and cost effective performance.

### The examiners

The program is mainly carried out by health visitors after a long period of training by the senior author (TBG). They form the main examining group, which is also occasionally staffed by Orthopaedic and General Medicine trainees and by Physiotherapists.

### Preparation for school screening

Two weeks before visiting a school the head-master was informed about the program by the screening staff and educational material was distributed. The parents were asked to sign a consent form and the pupils were asked to fill particular forms regarding their personal data. The program was performed once a week, during the school period (September to June).

### The examined children

Although the program started in 1997, accurate financial data is available since 2000. From January 2000 to May 2006, 6470 pupils aged 6–18 years old were screened for spinal deformities. Screening included both boys and girls.

### The measurements

Prior to examination the screening staff collected the filled forms and personal data for every pupil (date of birth, sex, stage of puberty, eye and hair color, height, weight, handedness) and socioeconomic parameters (parents' age, origin and profession) were recorded.

Pupils were examined in their physical educational environment to avoid any psychological impact, unnecessary time loss and transportation expenditures for them. Groups of 15 – 20 pupils were subsequently invited into the examination room. Boys and girls were examined in different teams. Children were wearing their trousers and a plain T-shirt. Firstly, the child was inspected in a standing position for possible deformities of the extremities, shoulder or pelvic asymmetry, lateral body inclination and asymmetry of the distance of the elbows from the trunk. Then, bending test in standing and sitting position was performed, by asking the child to freely bend forward hanging his hands to the ground, keeping his palms opposed, his feet together and his knees straight for the inspection of a possible hump. The angle of trunk inclination (ATI) was measured using the Pruijs scoliometer in the thoracic, thoracolumbar and lumbar spine.

Pupils who were found with a scoliometer reading ≥ 7° were referred for further evaluation at the scoliosis clinic. The children's parents were informed by a letter and an appointment was given. If the Orthopaedic surgeon confirmed the suspected scoliosis by physical examination, a standing posteroanterior roentgenogram of the entire spine was obtained and if necessary treatment was initiated.

### The assessed parameters

In order to calculate the direct cost of the screening program, numerous factors were taken into consideration, namely: a) the number of the examiners, b) the working hours, c) the examiners' salary (the wages before tax), d) the cost of transportation from the number of school visits, the mean kilometer distance and the mean fuel cost per kilometer and e) the cost of examination per child.

## Results

Between January 2000 and May 2006 twenty examiners were involved in the program. The hourly compensation as it draws from the Financial Department of the hospital was 6.80 € for trainee doctors, 6.70 € for the health visitors and 5.50 € for the physiotherapists in current prices.

During the year *2000*, 1227 children were screened by five health visitors, ten training doctors and five physiotherapists. Doctors and physiotherapists were contributed to the program occasionally. Working hours for each specialty were 104 for the doctors, 187 for health visitors and 83 for the physiotherapists. The entire cost for each specialty was calculated by multiplying the working hours by the hourly compensation and it was 707.20 € for trainee doctors, 1252.90 € for health visitors and 456.50 € for physiotherapists. The examiners' cost of transportation was calculated to be 31.2 €. The average cost per child for the year 2000 was 1.99 €, (Table 1).

**Table 1:** Estimation of the direct cost of "Thriasio" school screening program.

Year		**2000**	**2001**	**2002**	**2003**	**2004**	**2005**	**2006**
Number of examined children		**1227**	**1111**	**1026**	**1018**	**565**	**649**	**874**
No of examiners	HV	**5**	**2**	**3**	**2**	**2**	**2**	**2**
	TD	**10**			**2**	**2**	**1**	**1**
	PT	**5**	**2**					
Working hours	HV	**104**	**176**	**167**	**192**	**160**	**196**	**147**
	TD	**187**			**192**	**160**	**98**	**73**
	PT	**83**	**14**					
Examiners' compensation	HV	**1252,9**	**1179,2**	**1118,9**	**1186,4**	**1072**	**1313,2**	**978,2**
	TD	**707,2**			**1305,6**	**1088**	**666,4**	**496,4**
	PT	**456,5**	**77**					
Cost of transportation		**31,2**	**30**	**25,2**	**30**	**32,5**	**34**	**33,2**
Cost per child		**1,99**	**1,15**	**1,11**	**2,58**	**3,88**	**3,1**	**1,72**

Analysis of the number of the examiners, the working hours, the examiners' compensation, the cost of transportation and the cost of examination per child in each year of the studied period. All costs were evaluated in euros, in current prices. HV: Health Visitors, TD: Training Doctors, PT: Physiotherapists.

During the year *2001*, 1111 children were screened by two health visitors and two physiotherapists. Health visitors worked for 176 hours and physiotherapists for 14 hours. The total cost was 1179.20 € for health visitors and 77 € for physiotherapists. The cost of transportation was 30 €. The average cost per child for the year 2001 was 1.15 €, (Table 1).

During the year *2002*, 1026 children were screened by three health visitors, who worked for 167 hours. The total cost was 1118.90 €. The cost of transportation was 25.20 €. The average cost per child for the year 2002 was 1.11 €, (Table 1).

During the year *2003*, 1018 children were screened by two health visitors and two trainee doctors. Each specialty worked for 192 hours. The total cost was 1286.4 € for the health visitors and 1305.6 € for the doctors. The cost of transportation was 30 €. The average cost per child for the year 2003 was 2.58 €, (Table 1).

During the year *2004*, 565 children were screened by two health visitors and two trainee doctors. Each specialty worked for 160 hours. The total cost was 1072 € for the health visitors and 1088 € for the doctors. The cost of transportation was 32.5 €. The average cost per child for the year 2004 was 3.88 €, (Table 1).

During the year *2005*, 649 children were screened by two health visitors and one trainee doctor. Health visitors worked for 196 hours and the doctor for 98 hours. The total cost was 1313.2 € for the health visitors and 666.4 € for the doctor. The cost of transportation was 34 €. The average cost per child for the year 2005 was 3.1 €, (Table 1).

During the year *2006*, 874 children were screened by two health visitors and one trainee doctor. Health visitors worked for 147 hours and the doctor for 73 hours. The total cost was 978.2 € for the health visitors and 496.4 € for the doctor. The cost of transportation was 33.2 €. The average cost per child for the year 2006 was 1.72 €, (Table 1).

The average cost for the examination of each child for the above studied period (2000 – 2006) was calculated to be 2.04 €.

## Discussion

The purpose of school screening is to identify most or all the individuals with unrecognized idiopathic scoliosis [10]. School screening programs should also meet specific criteria. They should be rapid, accurate, low-cost, use tests which are easily reproducible and have low false positive and false negative results. [10].

School screening is a valuable tool for the identification of children with idiopathic scoliosis [11,14] although it is not a diagnostic process. Early detection allows the more progressing curves to be treated conservatively. Conservative treatment may alter the natural history of scoliosis [15-17]. In areas in which there are school screening programs fewer patients with idiopathic scoliosis ultimately require surgery [11,12,18].

Moreover, the results of school screening programs provide valuable data regarding the prevalence and the natural history of idiopathic scoliosis [10,12,19,20]. Considering that there are no sufficient epidemiological data in the literature for the prevalence of idiopathic scoliosis in several geographical areas and the natural history is not yet accurately predictable, we can assume that the school screening is not only an issue of early detection and decrease in the number of adolescents that will eventually experience operative treatment, but is also a priceless tool for research on scoliosis aetiology [21-32].

This paper analyzes the direct cost of the "Thriasio" school screening program. A realistic evaluation of both direct and indirect cost is not feasible and could result in inaccurate overestimation of the total cost as it might take into consideration many qualitative and subjective factors, such as the definition of scoliosis, the threshold for referrals for radiological evaluation, the indications for conservative and operative treatment, the cost to the society, the children's compliance, the decisions of the clinicians, the effectiveness of treatment and the impact on children's quality of life [13,19,33]. The negativists of school screening are implicating the increased indirect cost and the psychological impact on the child, which basically cannot be measured, to criticize these programs. However they are not discussing about the cost of family's and child's psychological stress when there is an untreated severe undiscovered curve, or the cost of the child's and family psychological stress when they will be in the operative room, or what is the feeling of an operated scoliotic with a rod in her back holding her straight. No one so far gave a frank answer on this issue. On the other hand, approaching the 'psychological' issue from a different viewpoint it could be stated that any negative impact of early diagnosis is intrinsically linked to an absence of effective therapies and failure of professional education and support; given appropriate resources to treat effectively (as with antibiotics in early stages of microbial infection, for example), as well as non-destructive ways to measure changes (i.e. not involving repetitive radiation exposure), 'psychological' damage could be considered as a moot point. Even in the absence of effective strategies and lack of support from the medical community available in some cultures, early on knowledge of the diagnosis provides patients with the tools to educate and help themselves.

The actions for prevention of a disease are the criterion of a human welfare oriented civilizations, even though this might cost some extra money. And the money exists.

Maybe the school screening and the techniques used for performing it are not yet perfected, but nothing is perfect.

There is no general consensus among economists as to what constitutes the indirect cost in a cost effectiveness analysis. Furthermore, there is no study which fully evaluates the direct and indirect cost of the school screening in the literature. Therefore, the economic information on screening for scoliosis which is available to decision-makers should mainly be based on studies of the direct cost of such programs.

Efforts were made by the examiners to minimize all the indirect costs. Emphasis was given to the training of the examiners in both their organization and clinical performance. Frequent courses, literature reviews and workshops were organized in order to produce efficient and reliable examiners [34-36]. The "Thriasio" school screening program is performed by trained health professionals (Orthopaedic trainee doctors, health visitors and physiotherapists). The team is examining the children with the use of a scoliometer in the forward bending standing and sitting position, which is a reproducible test with high sensitivity and high specificity [9,33]. By minimizing the false positive results [19] the program is more effective with fewer referrals and minor psychological impact on children and their families.

Table 1 reveals several important variances directly related to cost, such as the number of the examiners, the child per examiner ratio and the number of hours worked per child examined. Namely the number of examiners ranges from a high of twenty including 10 trainee doctors to a low of 3 utilizing Health Visitors only. The child per examiner ratio varies from a high of 437 to a low of 61. The exam in 2000 included 5 Physiotherapists which reduced to 2 Physiotherapists in 2001 and then none for the remaining 5 years. The number of hours worked per child examined ranged from .16 in 2002 to .57 in 2004 which is directly reflected in the cost per child examined in the latter year being 3.88 euro. The core of the examiners' group who were involved in the program were two experienced and well trained for three years by the senior author (TBG) health visitors who occasionally were supported by other health visitors, training doctors or physiotherapists, according to their interests and limited by the staff shortage at a given time. The main aim was and remains the continuation of our school screening program. The number of the examiners did not affect the number of hours worked per child examined. The variance in the number of hours worked per child was related to many independent factors such as the number of pupils in each school, their age, the available facilities for the undressing and dressing and the examination of the children at schools, or the necessity to complete some administrative work which was not prepared by the teachers prior to the visits. Therefore the model of estimation of the direct cost based on the hours worked per child examined, even thought appears to be more meaningful than solely focusing on the direct cost because it is more easily comparable to other programs regardless of the local economy, seems to be not applicable in all places due to the restrains of the above described independent factors.

All the administrative work with the schools and families, the training of the examiners, the examination at schools and the follow up evaluation by the Orthopaedic Surgeon were activities which did not surcharge the hospital, as they were performed within the working hours and were part of the duties of the involved health professionals. The present study introduces a model for running a school screening program which is inexpensive, because it is carried out in a voluntary basis by health professionals and does not cost extra money to the hospital. The "Thriasio" screening program is not funded by anyone!

Conservative treatment of idiopathic scoliosis is effective [37] and is definitely not as expensive as operative treatment. Idiopathic scoliosis prevalence in Greece is 2%. Nine hundred and eighty children will need conservative and 41 children will need operative treatment annually [35]. The average cost of a brace in Greece is 1000 €, whilst the cost for operative treatment for each child might be 30–35000 € (cost of preoperative imaging with CT and/or MRI, hospitalization, cost of implants and somatosensory evoked potentials used intraoperatively, complications etc). Early conservative treatment should prevent the need for much surgery and therefore is a cost-saving procedure avoiding the expenses of surgical treatment, loss of earnings and disability payments among other things [10].

The direct cost of "Thriasio" school screening program is comparable to other programs in Greece [28,31].

Dr. Bunnell's human oriented motivation is well described by reporting that "... we're not looking for the cheapest way to screen – we're looking for a better quality outcome for our patients..." [20]. The present study provides a model and a strong economical evidence for the continuation of the school screening programs in the future, for better quality outcome for our patients using a cheap way to screen.

## Definitions

### Direct costs

Costs that can be identified specifically with a particular sponsored project, an instructional activity or any other institutional activity, or that can be directly assigned to activities relatively easily with a high degree of accuracy. These direct costs include salaries, wages, fringe benefits, materials, supplies, equipment, travel, consulting, printing, telephone, and photocopying [38].

### Indirect costs (facilities and administrative costs)

Costs that are incurred for common or joint objectives and therefore cannot be identified readily and specifically with a particular sponsored project, an instructional activity, or any other institutional activity [38].

## Authors' contributions

TBG organized, realized and is the scientific director of the "Thriasio" school screening program, conceived the idea of the presented study, performed part of the literature review and contributed in drafting of the manuscript. EV participated in the school screening program, performed part of the literature review and contributed in manuscript drafting and in the interpretation of data. CM is the chief examiner of the "Thriasio" school screening program, collected, fed the database in SPSS software and analyzed the data. ODS performed part of the literature review and participated in the school screening program. All authors have read and approved the final manuscript.

## References

[B1] Dickson RA (1983). Scoliosis in the community. Br Med J.

[B2] Leaver JM, Alvik A, Warren MD (1982). Prescriptive screening for adolescent idiopathic scoliosis – a review of the evidence. International Journal of Epidemiology.

[B3] Morais T, Bernier M, Turcotte F (1985). Age- and sex-specific prevalence of scoliosis and the value of school screening programs. Am J Public Health.

[B4] Ziporyn T (1985). Scoliosis management now subject of numerous questions. JAMA.

[B5] Scoliosis Research Society (1986). Scoliosis A handbook for patients.

[B6] American Academy of Orthopaedic Surgeons. Position Statement (1993). School screening programs for early detection of scoliosis.

[B7] US Preventive Service Task Force (2004). Screening for Idiopathic Scoliosis in Adolescent Recommendation statement.

[B8] Burwell G (1988). The British Decision and Subsequent Events. Spine.

[B9] Williams JL (1988). Criteria for screening: Are the effects predictable?. Spine.

[B10] Renshaw TS (1988). Screening school children for scoliosis. Clin Orthop.

[B11] Montgomery F, Persson U, Benoni G, Willner S, Lindgern B (1990). Screening for scoliosis. A cost-effectiveness analysis. Spine.

[B12] Lonstein JE, Bjorklund S, Wanninger MH, Nelson RP (1982). Voluntary school screening for scoliosis in Minnesota. J Bone Joint Surg Am.

[B13] Torell G, Nordnall A, Nachemson A (1981). The changing pattern of scoliosis treatment due to effective screening. J Bone and Joint Surg Am.

[B14] Asher MA, Green P, Orrick J (1988). Scoliosis evaluation. Orthopedic Clinics of North America.

[B15] Grivas TB, Vasiliadis E, Chatziargiropoulos T, Polyzois VD, Gatos K (2003). The effect of a modified Boston brace with antirotatory blades on the progression of curves in Idiopathic Scoliosis. Aetiologic implications. Pediatric Rehabilitation.

[B16] Korovessis P, Kyrkos C, Piperos G, Soucacos PN (2000). Effects of thoracolumbosacral orthosis on spinal deformities, trunk asymmetry and frontal lower rib cage in adolescent idiopathic scoliosis. Spine.

[B17] Rowe DE, Bernstein SM, Riddick MF, Adler F, Emans JB, Gardner-Bonneau DA (1997). Meta-analysis of the efficacy of non-operative treatments for idiopathic scoliosis. J Bone Joint Surg Am.

[B18] Brooks HL, Azen SP, Gerberg E, Brooks R, Chan L (1975). Scoliosis: A prospective epidemiological study. J Bone Joint Surg Am.

[B19] Lonstein JE (1988). Why school screening for scoliosis should be continued. Spine.

[B20] Bunnel WP (2005). Selective screening for scoliosis. Clin Orthop Relat Res.

[B21] Grivas TB, Stavlas P, Koukos K, Samelis P, Polyzois B (2002). Scoliosis and Cavus Foot. Is there a Relationship? Study in Referrals, with and without Scoliosis, from School Screening. Stud Health Technol Inform.

[B22] Grivas TB, Samelis P, Pappa AS, Stavlas P, Polyzois D (2002). Menarche in Scoliotic and Nonscoliotic Mediterranean Girls. Is There Any Relation between Menarche and Laterality of Scoliotic Curves?. Stud Health Technol Inform.

[B23] Grivas TB, Daggas S, Polyzois B, Samelis P (2002). The double rib contour sign in lateral spinal radiographs. Aetiologic implications for scoliosis?. Stud Health Technol Inform.

[B24] Grivas TB, Samelis P, Chatziargiropoulos T, Polyzois D (2002). Study of the rib cage deformity in children with 10°–20° of Cobb angle late onset idiopathic scoliosis, using rib vertebra angles. Stud Health Technol Inform.

[B25] Grivas TB, Daggas S, Samelis P, Maziotou C, Kandris (2002). Lateral spinal profile in school-screening referrals with and without late onset idiopathic scoliosis 10°–20°. Stud Health Technol Inform.

[B26] Grivas TB, Arvaniti A, Maziotou C, Manesioti M, Fergadi A (2002). Comparison of body weight and height between normal and scoliotic children. Stud Health Technol Inform.

[B27] Korovessis P, Stamatakis M (1996). Prediction of Cobb angle with the use of a scoliometer. Spine.

[B28] Koukourakis I, Giaourakis G, Kouvidis G, Kivernitakis E, Blazos J, Koukourakis M (1997). Screening School Children for Scoliosis on the Island of Crete. J Spinal Disorders.

[B29] Smyrnis PN, Valavanis J, Voutsinas S, Alexopoulos A, Ierodiaconou M, Zorab PA, Siegler D (1980). Incidence of scoliosis in the Greek islands. Scoliosis 1979 – Based on the proceedings of the sixth symposium on scoliosis held at the Cardiothoracic Institute Brompton Hospital London on 17th and 18th September 1979.

[B30] Smyrnis PN, Valavanis J, Alexopoulos A, Siderakis G, Gianestras NJ (1979). School screening for scoliosis in Athens. J Bone Joint Surg Br.

[B31] Soucacos NP, Soucacos KP, Zacharis KC, Beris AE, Xenakis TA (1997). School-Screening for Scoliosis. A prospective epidemiological study in Northwestern and central Greece. J Bone Joint Surg Am.

[B32] Soucacos PN, Zacharis K, Gelalis J, Soultanis K, Kalos N, Beris A, Xenakis T, Johnson EO (1998). Assessment of curve progression in idiopathic scoliosis. Eur Spine J.

[B33] Ashworth MA, Hancock JA, Ashworth L, Tessier KA (1988). Scoliosis screening: an approach to cost/benefit analysis. Spine.

[B34] Grivas TB (2000). School Screening for Scoliosis.

[B35] Grivas TB, Koukos K, Koukou U, Maziotou CH, Polyzois BD (2002). The incidence of idiopathic scoliosis in Greece. Stud Health Technol Inform.

[B36] Grivas TB, Samelis P, Polyzois BD, Giourelis B, Polyzois D (2002). School Screening in the heavily industrialized. Is there any role of industrial environmental factors in Idiopathic Scoliosis prevalence?. Stud Health Technol Inform.

[B37] Lonstein JE (1988). Natural history and school screening for scoliosis. Orthopedic Clinics of North America.

[B38] http://www.sps.arizona.edu/postaward/directindirectcostpolicy.htm.

